# Higher education responses to COVID-19 in the United States: Evidence for the impacts of university policy

**DOI:** 10.1371/journal.pdig.0000065

**Published:** 2022-06-23

**Authors:** Brennan Klein, Nicholas Generous, Matteo Chinazzi, Zarana Bhadricha, Rishab Gunashekar, Preeti Kori, Bodian Li, Stefan McCabe, Jon Green, David Lazer, Christopher R. Marsicano, Samuel V. Scarpino, Alessandro Vespignani

**Affiliations:** 1 Network Science Institute, Northeastern University, Boston, United States of America; 2 Laboratory for the Modeling of Biological and Socio-Technical Systems, Northeastern University, Boston, Massachusetts, United States of America; 3 Biosecurity and Public Health Group, Los Alamos National Laboratory, Los Alamos, New Mexico, United States of America; 4 College of Engineering, Northeastern University, Boston, Massachusetts, United States of America; 5 College of Professional Studies, Northeastern University, Boston, Massachusetts, United States of America; 6 Shorenstein Center on Media, Politics and Public Policy, Harvard University, Massachusetts, Boston, United States of America; 7 Educational Studies Department, Davidson College, Davidson, North Carolina, United States of America; 8 College Crisis Initiative, Davidson College, Davidson, North Carolina, United States of America; 9 Vermont Complex Systems Center, University of Vermont, Burlington, Vermont, United States of America; 10 Santa Fe Institute, Santa Fe, United States of America; Tsinghua University, CHINA

## Abstract

With a dataset of testing and case counts from over 1,400 institutions of higher education (IHEs) in the United States, we analyze the number of infections and deaths from SARS-CoV-2 in the counties surrounding these IHEs during the Fall 2020 semester (August to December, 2020). We find that counties with IHEs that remained primarily online experienced fewer cases and deaths during the Fall 2020 semester; whereas before and after the semester, these two groups had almost identical COVID-19 incidence. Additionally, we see fewer cases and deaths in counties with IHEs that reported conducting any on-campus testing compared to those that reported none. To perform these two comparisons, we used a matching procedure designed to create well-balanced groups of counties that are aligned as much as possible along age, race, income, population, and urban/rural categories—demographic variables that have been shown to be correlated with COVID-19 outcomes. We conclude with a case study of IHEs in Massachusetts—a state with especially high detail in our dataset—which further highlights the importance of IHE-affiliated testing for the broader community. The results in this work suggest that campus testing can itself be thought of as a mitigation policy and that allocating additional resources to IHEs to support efforts to regularly test students and staff would be beneficial to mitigating the spread of COVID-19 in a pre-vaccine environment.

## Author summary

The ongoing COVID-19 pandemic has upended personal, public, and institutional life and has forced many to make decisions with limited data on how to best protect themselves and their communities. In particular, institutes of higher education (IHEs) have had to make difficult choices regarding campus COVID-19 policy without extensive data to inform their decisions. To better understand the relationship between IHE policy and COVID-19 mitigation, we collected data on testing, cases, and campus policy from over 1,400 IHEs in the United States and analyzed the number of COVID-19 infections and deaths in the counties surrounding these IHEs. Our study found that counties with IHEs that remained primarily online experienced fewer cases and deaths during the Fall 2020 semester—controlling for age, race, income, population, and urban/rural designation. Among counties with IHEs that did return in-person, we see fewer deaths in counties with IHEs that reported conducting any on-campus testing compared to those that reported none. Our study suggests that campus testing can be seen as another useful mitigation policy and that allocating additional resources to IHEs to support efforts to regularly test students and staff would be beneficial to controlling the spread of COVID-19 in the general population.

## Introduction

Younger adults account for a large share of SARS-CoV-2 infections in the United States, but they are less likely to become hospitalized and/or die after becoming infected [1–5]. Mitigating transmission among this population could have a substantial impact on the trajectory of the COVID-19 pandemic [2]; younger adults typically have more daily contacts with others [6–8], are less likely to practice COVID-19 mitigation behaviors [9, 10], are more likely to have have jobs in offices or settings with more contacts with colleagues [11], and travel at higher rates [12–14]. Additionally, in the United States, over 19.6 million people attend institutes of higher education (IHEs; i.e., colleges, universities, trade schools, etc.) [15], where students often live in highly clustered housing (e.g. dorms), attend in-person classes and events, and gather for parties, sporting events, and other high-attendance events.

Because of this, the COVID-19 pandemic presented a particular challenge for IHEs during the Fall 2020 semester [16–21]. On the one hand, bringing students back for on-campus and in-person education introduced the risk that an IHE would contribute to or exacerbate large regional outbreaks [22–30]; on the other hand, postponing students’ return to campus may bring economic or social hardship to the communities in which the IHEs are embedded [31–34], since IHEs are often large sources of employment for counties across the United States. As a result, IHEs instituted a variety of “reopening” strategies during the Fall 2020 semester [35–44]. Among IHEs that brought students and employees back to campus—either primarily in person or in a “hybrid” manner—we see different approaches to regularly conducting (and reporting) COVID-19 diagnostic testing for students, faculty, and staff throughout the semester.

Most of these policies were designed to minimize spread within the campus population as well as between the IHE and the broader community. These policies include testing of asymptomatic students and staff, isolating infectious students, quarantining those who were potentially exposed through contact tracing, extensive cleaning, ventilation, mask requirements, daily self-reported health assessments, temperature checks, and more [45, 46]. As with much of the COVID-19 pandemic [47], these policies were often instituted in a heterogeneous manner, with varying levels of severity [37], which makes studying their effects both important and challenging. Studying the various differences between these policies is made even more difficult because of the lack of a centralized data source and standardized reporting style. On top of that, counties with IHEs represent a wide range of demographics (age, income, race, etc.) [48], which must be accounted for when comparing any policies, since these factors have known associations with an individual’s likelihood of hospitalization or death [49, 50].

**Fig 1:**
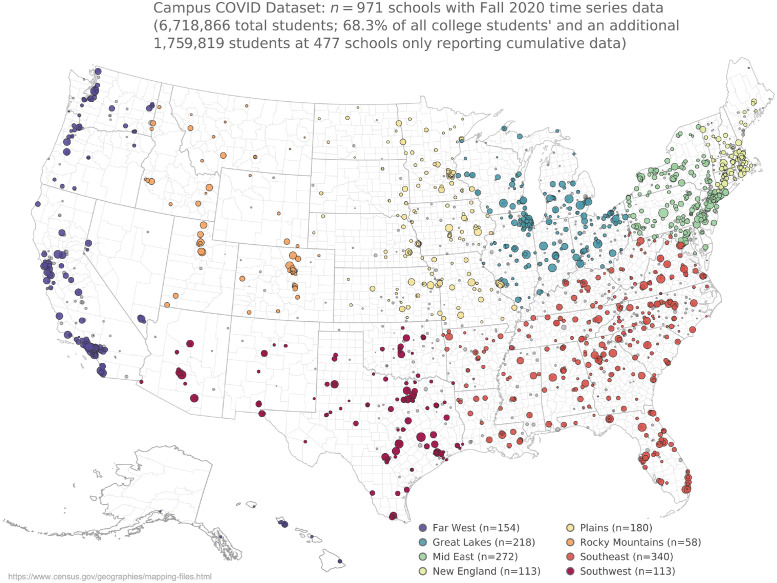
Description of the Campus COVID Dataset. Map of the 1,448 institutes of higher education included in the Campus COVID Dataset. The dataset includes semester-long time series for 971 institutes of higher education (see S1 Text for several examples), in addition to 477 that have cumulative data only (i.e. one sum for the total testing and/or case counts for the Fall 2020 semester). County and state boundary maps downloaded from the United States Census TIGER/Line Shapefiles [52].

Many IHEs developed and maintained “COVID dashboards” [51] that update the campus community about the number of COVID-19 cases reported/detected on campus and, if applicable, the number of diagnostic tests conducted through the IHE. Here, we introduce a dataset of testing and case counts from over 1,400 IHEs in the United States (Fig 1), and we use this dataset to isolate and quantify the impact that various IHE-level policies may have on the surrounding communities during the Fall 2020 semester (August to December, 2020). After a matched analysis of statistically similar counties, we show that counties with IHEs that reopened for primarily in-person education had a higher number of cases and deaths than counties with IHEs that did not. Among IHEs that did allow students back on campus, we see fewer cases and deaths on average if the county contains IHEs that conduct on-campus COVID testing. We further examine this result by focusing on data from IHEs in Massachusetts, where we find that cities with IHEs that test more also have fewer average cases per capita. This pattern holds in spite of the number of cases detected among members of the campus community. These results point to a benefit of large-scale, asymptomatic testing of the campus community (students, faculty, staff, etc.), which can be especially important in regions without (or with fewer) local mitigation policies in place.

## Results

Throughout this section, we aim to make two broad comparisons. First, we look at different approaches for how IHEs reopened for the Fall 2020 semester (early August through early December, 2020). We compare COVID-19 outcomes between counties with IHEs where students returned to fully or *primarily in-person* education and counties with IHEs that remained fully or *primarily online*. IHE reopening status is based on data from [37]. We group the categories of “fully online” and “primarily online” into “primarily online” and do the same for fully/primarily in-person; schools listed as “hybrid” are not included in this comparison but present interesting avenues for future research. Second, we quantify the benefits of IHE-affiliated testing by comparing COVID-19 outcomes between counties with IHEs that *reported any campus testing* and counties with IHEs that *reported no campus testing*. Testing data are from the Campus COVID Dataset [53] (see Data & methods section) and were collected manually through the COVID dashboards of over 1,400 IHEs. To perform the two main comparisons above, we carefully match groups of counties in order to avoid potential confounding effects of the underlying demographics of the counties’ populations.

### Comparing counties with similar demographics

#### Constructing groups of counties

COVID-19 has had a disproportionate impact on older populations, and we see especially high death rates in regions with more congregate senior living and long-term care facilities [54, 55]. On the other hand, in regions with more young people (i.e., “college towns”—or, here, college counties) experienced relatively fewer hospitalizations and deaths [55]. This means that care should be taken when comparing averages between groups of counties, and prior to creating the groups, we must attempt to match the underlying demographics of the groups as much as possible.

This becomes an optimization problem: there are over 1,238 different counties with IHEs in our dataset. We want to separate them into two groups of counties, *A* and *B*, that are roughly equivalent in size and that consist of counties with as similar distributions of demographics as possible. For example, here we create a group of counties with IHEs that returned primarily in-person (*n*_*A*_ = 393 total) and a group of counties with IHEs that remained primarily online (*n*_*B*_ = 449 total) during the Fall 2020 semester. In our case, a key variable we will optimize over, *x*, is the percent of county population enrolled at IHEs full-time. The reason for this is intuitive: we want to compare *college counties*, which we define based on the fraction of IHE students among the total population. However, it is not necessarily obvious what value this threshold *x* should take (e.g. is a college county one where *x* = 0.1% of the total population is a full-time IHE student? 1.0%? 10%? etc.), so we use an optimization technique in order to select the value for *x*.

This procedure iteratively measures the Jensen-Shannon divergence (JSD) between the distributions of demographic variables between the two groups. The JSD between two distributions, *P* and *Q*, is $JSD\left(P\|Q\right)=\frac{1}{2}D\left(P\|M\right)+\frac{1}{2}D\left(Q\|M\right)$, where $M=\frac{1}{2}\left(P+Q\right)$ and *D* refers to the Kullback-Leibler Divergence; higher JSD values indicate higher dissimilarity. As an example, we know that age is a key variable in determining COVID-19 outcomes [1], and as such, we want the average age (and distribution of ages) of the two groups to resemble one another as much as possible (i.e., select grouping that minimizes *JSD*(*Age*_1_||*Age*_2_)). In total, we focus on five county demographic variables: age, race, income, total population, and urban-rural code. Since the JSD captures the extent to which pairs of distributions are different, we want to find the value for *x* that minimizes the total JSD (see S1 Text for more details and Fig A in S1 Text for the minima of the five variables).

Following this optimization procedure, we determined the threshold to be 3.68%. That is, in order for a county to be included in the “primarily in-person” group (or, analogously, the “primarily online” group), the total number of full-time students attending “primarily in person” IHEs must exceed 3.68% of the total population. The specific value for this threshold may appear ad hoc or arbitrary, but importantly, the two groups we are left with are highly similar along our key variables of interest. See S1 Text where we describe this procedure in depth and show how similar the distributions of demographic variables between the resulting groups are (Figs B and C in S1 Text).

In summary, we create two groups of college counties—those with IHE students who returned to the Fall 2020 semester primarily in-person and those with IHE students who remained primarily online. To do this, we had to choose what constituted a “college county”—we determined that this should be based on the percent of IHE students in a county’s total population, *x*. At the same time, we wanted to ensure statistical and demographic similarity between the populations in each group. In the end, we selected *x* = 3.68%, the value that minimized the total JSD between the distributions of interest between the two groups.

#### Fall 2020 reopening status: In-person vs. online education

**Fig 2:**
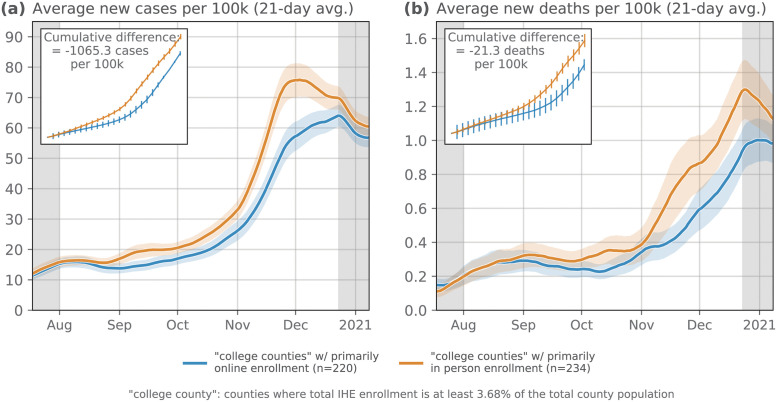
Counties with IHEs categorized as in-person vs. online for Fall 2020. Here, we compare the average **(a)** new cases and **(b)** new deaths per 100,000 in counties with IHEs that were categorized as “primarily in-person” vs. “primarily online” for the Fall 2020 semester. IHEs classified with “hybrid” reopening strategy were not included in this comparison as there is a great deal of heterogeneity in what constitutes a “hybrid” reopening. IHE reopening data is from [37]. (Ribbons: 95% confidence interval).

With this grouping, we now compare the average new cases and new deaths per 100,000 between the two groups (Fig 2). By minimizing the demographic variability between the “in-person” counties and the “online” counties, we get closer to addressing the questions surrounding the effects of IHE policy on the broader community. In Fig 2a, we see that during July and August the number of new cases per 100,000 was almost identical for the in-person and online counties. By the end of August (i.e., the start of the Fall 2020 semester), we begin to see these two curves diverge; college counties with primarily in-person enrollment report more new cases per 100,000 on average for the remainder of 2020, a gap that narrows shortly after the Fall 2020 semester ends.

We see the same trend—but lagged by about four weeks—when comparing the average new deaths per 100,000 between the two groups of counties. These analyses, even after controlling for several potentially confounding demographic variables, highlight clear differences in COVID outcomes based on IHE reopening policy. In S1 Text, we also show how the matching procedure used here excludes other potentially confounding *spatial* variables as well. For example, counties that were hit early and hard by COVID-19 in March and April of 2020 (e.g. counties in New York City, greater Boston area, etc.) are already not included in these averages (see a visualization of the included counties in Fig C in S1 Text).

#### Quantifying the benefits of IHE-affiliated testing

The extent to which IHEs tested their students and employees for COVID-19 varied substantially: some schools focused their limited testing resources on only testing *symptomatic* individuals while others developed a strict and massive testing program that required frequent (e.g. weekly) asymptomatic testing. Because of this heterogeneity, we sought the simplest distinction for comparing groups of counties; we split the “primarily in person” counties from the Fall 2020 Reopening Status section into two groups of counties: those with IHEs that reported conducting *any* COVID-19 tests on campus and those that reported *none*. Of the *n* = 234 “primarily in person” counties from the previous section, the Campus COVID Dataset includes data from *n* = 144 counties. Often, when IHEs do not administer any on-campus tests, they have a form for students and staff to self-report results from external testing providers (e.g. pharmacies, health clinics, etc.). In order to classify an IHE as “non-testing” we sought out official documentation on the IHE’s websites, though a key limitation of this approach is that an IHE could have been conducting testing without posting updates to their websites.

**Fig 3:**
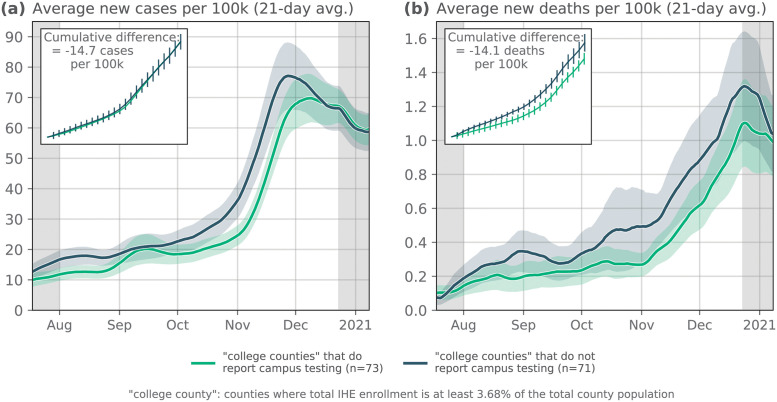
Comparing counties with IHEs that reported any vs. zero COVID-19 tests. As in Fig 2, we compare the average **(a)** new cases and **(b)** new deaths per 100,000 in counties with IHEs reported conducting any COVID-19 tests vs. counties with IHEs that reported no tests. Note: if there are multiple IHEs in a single county, we sum together the total number of tests between all IHEs. (Ribbons: 95% confidence interval).

Counties with IHEs that reported conducting a nonzero number of tests saw, on average, fewer reported cases and deaths (Fig 3). Notably, we see an increase in the number of cases per 100,000 on average in early September 2020 among counties with IHEs that do report testing (i.e., the campus testing is working as designed—detecting cases in the campus population; Fig 3a); this same increase in reported cases *does not* appear among counties with IHEs that do not report testing. This suggests that the return-to-campus surges that were being detected in IHEs that report testing may have occurred but remained undetected or under-reported in counties without IHE-affiliated testing. This suggestion is in part corroborated by the increase in deaths in the middle of October among counties without IHE testing, which does not appear to follow a commensurate increase in case counts (Fig 3b).

Importantly, while the two groups of counties used in this comparison were relatively balanced with respect to demographic variables, they are not formed based on information about differences in county-level mitigation policies that may have been active in the counties during this time period; this is in part due to relatively sparse data around county-specific policies (though there are data sets that report some county-level policies; see [56]). Perhaps more importantly, however, we do not include these data here because of the difficulty in standardizing the implementation and enforcement of specific policies (e.g. see [47] to look at the impact of heterogeneous policies across regions). Despite that, it is conceivable that IHEs that report more campus testing are *also* embedded in counties with more stringent mitigation policies in place; as such, even though we observe on average fewer cases and deaths in counties with IHEs that report campus testing, it would be incomplete to assume that IHE testing is the only reason for these differences. To get at addressing these points, we conducted additional analyses in S1 Text where we used state-level policy data [57] to quantify the effect of IHE testing policy while controlling for a number of county-level demographic variables as well as the number of active mitigation policies in place. Here again, we see significantly fewer deaths per 100,000 in counties with IHEs that conduct campus testing (Table 1).

**Table 1:** Generalized Linear Model (GLM) Regression: Predicting COVID-19 deaths, at *t* + 38 days. Regression table under a Negative Binomial model. See Table C and Fig D in S1 Text for descriptions of variables. Standard errors were adjusted for clustering at the county level. Coefficients in **bold** are statistically significant at the 95% confidence level.

**Dep. Variable**:	new deaths per 100k, at *t* + 38 days	**No. Observations**:	55842
**Model**:	GLM	**Df Residuals**:	55830
**Model Family**:	NegativeBinomial	**Df Model**:	11
**Link Function**:	log	**Scale**:	1.0000
**Method**:	IRLS	**Log-Likelihood**:	-62440.
**No. Iterations**:	8	**Deviance**:	31331.
**Covariance Type**	cluster (county)	**Pearson chi2**:	4.09e+04

	**coef**	**std err**	**z**	**P>|z|**	**[0.025**	**0.975]**
**average temperature (°Celsius)**	**-0.0391**	0.003	-14.917	0.000	-0.044	-0.034
**urban/rural code (1 if ∈ {4, 5, 6} else 0)**	0.1014	0.070	1.442	0.149	-0.036	0.239
**log(population density)**	-0.0168	0.033	-0.513	0.608	-0.081	0.047
**log(median income)**	**-0.4722**	0.121	-3.908	0.000	-0.709	-0.235
**2020 voting behavior (% two-party vote)**	**0.0124**	0.003	3.860	0.000	0.006	0.019
**log(population over 60 per 100k)**	**0.4896**	0.121	4.042	0.000	0.252	0.727
**log(IHE fulltime enrollment per 100k)**	**-0.1028**	0.049	-2.084	0.037	-0.199	-0.006
**log(IHE fulltime enrollment per 100k (online))**	0.0088	0.010	0.890	0.374	-0.011	0.028
**log(IHE fulltime enrollment per 100k (in person))**	0.0120	0.009	1.283	0.199	-0.006	0.030
**stringency index (OxCGRT)**	**-0.0178**	0.004	-4.171	0.000	-0.026	-0.009
**log(county new tests per 100k)**	**0.2663**	0.046	5.823	0.000	0.177	0.356
**log(IHE new tests per 100k)**	**-0.0382**	0.015	-2.558	0.011	-0.068	-0.009

Lastly, the grouping selected here (no reported testing vs. any reported testing) does not provide insights into the ideal amount of IHE testing needed to manage campus outbreaks. However, we examine this question in the following section, where we group cities in Massachusetts based on the amount of IHE testing, as opposed to simply whether they have IHE testing or not. Future work will examine whether there are optimal trade-offs between testing volume, cost of testing, levels of local transmission, and community demographics.

### Case study: Higher education in Massachusetts

According to the data collected in this work, IHEs in Massachusetts administered more COVID-19 tests to students and staff, on average, than most other states. As such, the Campus COVID Dataset includes time series data for 56 IHEs during both the Fall 2020 and Spring 2021 semesters. Additionally, the Massachusetts Department of Health releases weekly data about testing and case counts at the *city* level (total of *n* = 351 cities instead of *n* = 15 counties) [59]. In this section, we analyze Massachusetts as an informative case study about the role that IHE-affiliated testing may play in a community’s response to COVID-19.

**Fig 4:**
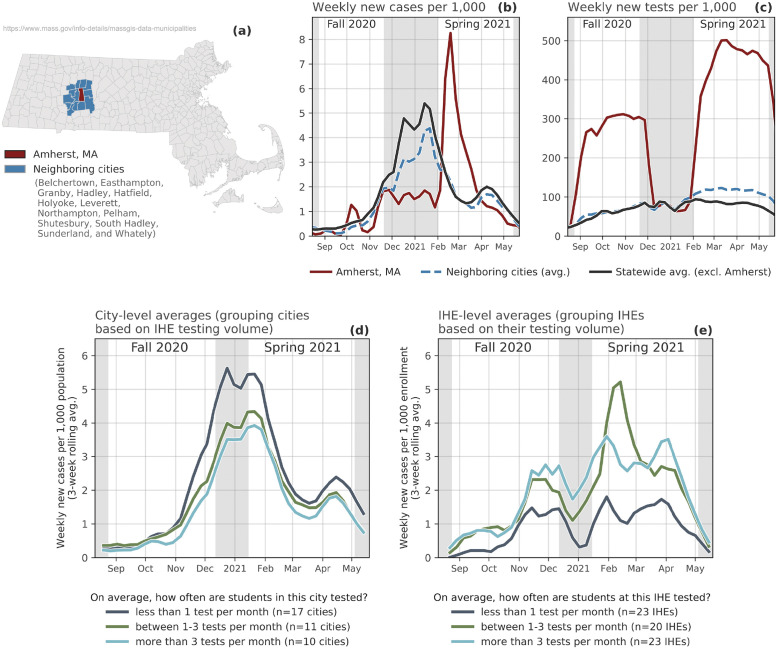
Case Study: COVID-19 in Massachusetts college cities. Top: Highlighting testing and case counts in and around Amherst, Massachusetts. **(a)** Map of Massachusetts cities; in this map, the city of Amherst is red and the surrounding cities are colored blue. **(b)** Time series of weekly new cases per 1,000 in: Amherst, the surrounding cities, and the rest of Massachusetts. **(c)** Time series of weekly new tests per 1,000 in: Amherst, the surrounding cities, and the rest of Massachusetts. Bottom: Comparing outcomes of cities and IHEs with more/less IHE-affiliated testing. **(d)** City-level average weekly new cases per 1,000, grouped by cities with IHEs that test students on average fewer than once a month, between one and three times a month, and over three times a month (Note: we sought out city-level data for COVID-19 deaths, but the state does not report these). **(e)** IHE-level average weekly new cases, grouped by IHEs that test students on average fewer than once a month, between one and three times a month, and over three times a month. Municipality boundary map downloaded from MassGIS (Bureau of Geographic Information) [58].

In February, 2021—at the start of the Spring semester—the University of Massachusetts, Amherst (UMass Amherst) experienced a large COVID-19 outbreak among the campus community. Throughout the Fall 2020 semester, UMass Amherst followed a campus testing regimen that required frequent testing of on-campus students and staff; as a result, the city of Amherst’s average number of tests per 1,000 residents was far higher than that of other cities in Massachusetts (Fig 4c). After the Fall 2020 semester ended, the overall testing volume in Amherst sharply declined since the number of students on campus decreases during December and January; the timing of this decline coincided with large *increases* in COVID-19 cases both regionally and statewide (Fig 4b & 4c). However, as neighboring cities to Amherst began to report a surge in cases during this period (Fig 4b), the city of Amherst did not report a commensurate rise in cases. It is possible that there were simply not as many cases in Amherst during December and January, but because there was such a large decrease in the amount of tests conducted during that period, it is also possible that there were some infections that remained undetected.

Either way, when students returned to campus in January, they returned to a city with a testing rate that was lower than it had been in late November, 2020. During the first few weeks of the Spring 2021 semester, UMass Amherst reported almost 1,000 new infections among students and staff, one of the largest outbreaks in the country at that time [60]. It is difficult to know whether the UMass Amherst outbreaks were primarily the result of importation from different areas following students’ return to campus or whether the returning students instead became infected following interactions with Amherst residents (or both). Regardless the source of these cases in Amherst, what happened *after* the February surge highlights the role that IHEs can have in local mitigation; testing volume in Amherst increased dramatically during the Spring 2021 semester, students who tested positive were strictly isolated, and on-campus restrictions of activities were put in place [61]. There indeed was a large outbreak, but without a robust on-campus testing protocol, the scale of this citywide outbreak might have grown even larger.

The example of UMass Amherst is a useful case study for highlighting a broader trend among Massachusetts cities with IHEs—a trend that largely mirrors the results in the previous section on IHE testing. In Fig 4d we show the average new cases per 1,000 for cities with IHEs that test their students an average of a) less than once per month, b) between 1–3 times per month, and c) more than three times per month; on average, cities with IHEs that conduct *more* tests also have *fewer* new cases. This pattern does not hold when only looking at on-campus cases from IHEs (as opposed to citywide cases); instead, we see that IHEs that test less also report fewer cases (Fig 4e). This trend may emerge because low-testing IHEs are not conducting enough tests to detect the true number of cases on campus, though proving this definitively is almost impossible without detailed contact tracing and/or retrospective antibody testing.

In the end, the resolution and completeness of the data for IHEs in Massachusetts give us an even more detailed look at the relationship between campus testing and new infections in the communities surrounding IHEs. Moving forward, improving the collection and reporting of this data nationwide will be crucial for our continued response to the COVID-19 pandemic.

## Discussion

The COVID-19 pandemic required governments and organizations to implement a variety of non-pharmaceutical interventions (NPIs) often without a thorough understanding of their effectiveness. Policy makers had to make difficult decisions about which policies to prioritize. While a body of literature has emerged since the beginning of the pandemic about measuring the effectiveness of NPIs [62–65], to date there have been no studies that attempt to measure the effectiveness of campus testing systematically nation wide. This study sheds light on this topic by directly measuring the impact of campus testing on county level COVID-19 outcomes. We collected data from 1,448 colleges and universities across the United States, recording the number of tests and cases reported during the Fall 2020 semester; by combining this data with standardized information about each school’s reopening plan, we compared differences in counties’ COVID-19 cases and deaths, while controlling for a number of demographic variables.

We used an entropy minimization approach to create two groups of counties that were as similar to demographic variables of interest (e.g., age, income, ethnicity) as possible in order to minimize confounding. The resulting groups had a similar number of counties per group, were spatially heterogeneous, and did not ultimately include counties from regions that experienced early surges in March, 2020 (e.g., counties in New York City, etc; see S1 Text), which could have confounding effects. When looking at county COVID-19 outcomes, our results shows that COVID-19 outcomes were worse in counties with IHEs that report no testing and in counties where IHEs returned to primarily in-person instruction during the Fall 2020 semester. These findings support the CDC recommendation to implement universal entry screening before the beginning of each semester and serial screening testing when capacity is sufficient [66] and are in line with smaller scale, preliminary results from other studies [67, 68]. While this study does not look at optimal testing strategies, it offers evidence for the protective effect of campus testing in any form and reopening status on county COVID-19 outcomes.

The COVID-19 pandemic highlighted the importance of data standardization for understanding the impact of the virus but also in to inform response, resource allocation, and policy. While much attention has been given to this topic for data reported by healthcare and public health organizations, little attention has been given for COVID-19 case and testing data reported by IHEs. A significant portion of the effort undertaken by this study was spent compiling and standardizing the data across IHEs nationwide. In the cases where IHEs did report campus testing data, the ease of access varied widely and oftentimes different metrics for cases and testing were reported out. For example, some IHEs would report only active cases, cumulative cases, or number of isolated individuals. Similarly, sometimes there would be no distinction between types of test given or temporal information on when the test was given. In their campus testing guidance [66], the CDC should also include recommendations on data standards and reporting formats.

While COVID-19 cases in the United States are lower than the peak in January 2021 and 2022, concerns remain around lingering outbreaks caused by new variants emerging, ongoing transmission in the rest of the world, vaccine hesitancy, and the possibility of waning effectiveness of the current vaccines [69, 70]. In regions like the Mountain West and South at the time of writing, vaccination rates remain disproportionately low among younger adults and the general population when compared to nation wide averages [71]. States in these same regions are also disproportionately represented among the states with the lowest IHE testing in our data set. Heterogeneity in vaccine uptake—and policy response broadly—makes it challenging to disentangle the effectiveness of any one specific policy response. On the one hand, further data collection on policy compliance (e.g. through online or traditional survey methods, digital trace data collection, etc.) may help to elucidate specific effects of different policies. On the other hand, because most of the current study focused on a period *before* widespread vaccine availability (and little impact of more transmissible SARS-CoV-2 variants), the Fall 2020 semester may in fact have been an ideal time to pose the questions in this work. In sum, given the number of younger adults enrolled in IHEs, the increased mobility and international nature of this population, and the fact that this population is less likely to practice COVID-19 mitigation behaviors, campus testing represents another effective control policy that IHEs and counties should consider to continue keeping COVID-19 incidence low.

## Data & methods

### Data collection and sources

County-level case data are from the COVID-19 Data Repository by the Center for Systems Science and Engineering (CSSE) at Johns Hopkins University [72]. County-level population and demographic data are from the 2018 American Community Survey (ACS) [73]. Weekly data for testing and case counts in Massachusetts cities are from the Massachusetts Department of Public Health [59]. Data about IHEs—including the number of full-time students and staff, campus location, institution type, etc.—come from the Integrated Postsecondary Education Data System (IPEDS) via the National Center for Education Statistics [74].

Data about individual IHEs’ plans for returning to campus (i.e., online only, in-person, hybrid, etc.) come from the College Crisis Initiative at Davidson College [37]. This dataset classifies IHEs based on the following categories, which we use to create three broader categories (in parentheses): “Fully in person” (primarily in-person), “Fully online, at least some students allowed on campus” (primarily online), “Fully online, no students on campus” (primarily online), “Hybrid or Hyflex teaching” (hybrid), “Primarily online, some courses in person” (primarily online), “Primarily in person, some courses online” (primarily in person), “Primarily online, with delayed transition to in-person instruction” (primarily online), “Professor’s choice” (hybrid), “Simultaneous teaching” (hybrid), “Some of a variety of methods, non-specific plan” (hybrid). We did not include “hybrid” IHEs in our analyses here, but they remain an interesting avenue for future research, which we strongly encourage using the Campus COVID Dataset.

### The campus COVID dataset

The Campus COVID Dataset was collected through a combination of web scraping, manual data entry, or communication with administrators at IHEs. In sum, the process involved collecting thousands of URLs of the COVID-19 dashboards (or analogous website) of each of over 4,000 IHEs, which we then used for manual data collection, inputting time series of case counts and testing volume between August 1 and December 16, 2020. The data for each IHE is stored in its own Google Sheet (indexed by a unique identifier, its ipeds_id), the URL of which is accessible through a separate Reference sheet. For full details on the data collection process, see S1 Text.

### Statistical controls for mitigation policies

While the two groups of counties—the “primarily in-person” vs. “primarily online” counties—are broadly similar across demographic categories (Fig B in S1 Text), there could still be underlying differences between the two groups that influence their different COVID-19 outcomes. For example, this could happen if the two groups differed in the extent to which they enacted mitigation policies (i.e., if there were a common variable influencing whether a given county introduced mitigation policies as well as whether IHEs in the county remained primarily online vs. in-person during the Fall 2020 semester). There are a number of possible sources of this variability, ranging from differences in population density [75], to differences in messaging from political leaders [76]. In the model below, we include the data about voting patterns in the 2020 presidential election in order to control for potential biases arising from differences in political behavior at the county level.

To control for potential biases arising from differences in local mitigation policies, we assigned each county to an “active mitigation policies” score based on policy tracking data from the Oxford COVID-19 Government Response Tracker [57]. These are daily time series data indicating whether or not a number of different policies were active on each day for a given state. Not only does this dataset list the presence or absence of a given policy, it also includes information about the severity (e.g. restrictions on gatherings of 10 people vs. restrictions on gatherings of 100 people, or closing all non-essential workplaces vs. closing specific industries, etc.). From these indicator variables, Hale et al. (2021) define a summary “stringency index” that characterizes the daily intensity of the mitigation policies that a given region is undergoing over time. We include this “stringency index” variable in an Generalized Linear Model regression to quantify the extent to which this time series of policy measures—along with data about IHE testing and enrollment policy, demographic data about the county itself, and average temperature—predicts COVID-19-related deaths (Table 1). After controlling for the variables above, we continue to see a significant negative association between the amount of IHE testing conducted in a county and COVID-19-related deaths, with a 38-day lag. Model specification and further details about the construction and interpretation of the model can be found in S1 Text.

### Citation diversity statement

Recent work has quantified bias in citation practices across various scientific fields; namely, women and other minority scientists are often cited at a rate that is not proportional to their contributions to the field [77–84]. In this work, we aim to be proactive about the research we reference in a way that corresponds to the diversity of scholarship in public health and computational social science. To evaluate gender bias in the references used here, we obtained the gender of the first/last authors of the papers cited here through either 1) the gender pronouns used to refer to them in articles or biographies or 2) if none were available, we used a database of common name-gender combinations across a variety of languages and ethnicities. By this measure (excluding citations to datasets/organizations, citations included in this section, and self-citations to the first/last authors of this manuscript), our references contain 12% woman(first)-woman(last), 21% woman-man, 22% man-woman, 38% man-man, 0% nonbinary, 4% man solo-author, 3% woman solo-author. This method is limited in that an author’s pronouns may not be consistent across time or environment, and no database of common name-gender pairings is complete or fully accurate.

## Supporting information

S1 Text Supporting information. **Table A: Current status of the Campus COVID Dataset**. In total, the Campus COVID Dataset includes data about more than 1,400 IHEs. To collect these data, we searched among over 2,719 IHEs; approximately 40% of these are IHEs with data that we could not find (because the IHE does not collect self-reported positive tests and/or does not conduct campus testing, etc.) or with data that we believe exists but was not being shared publicly by the IHE. There are over 971 IHEs with time series of testing and/or case counts for the Fall 2020 semester. If an IHE reported only cumulative testing or case counts, we classify it as “cumulative only”. **Table B: Example template for inputting data**. Each IHEs in the Campus COVID Dataset has a unique URL that leads to a dataframe with this structure. For each date that the IHE reports a number of new cases (“positive_tests” above) or new tests administered (“total_tests” above), we input that value in its corresponding row. For IHEs that report testing and case counts weekly, we insert the data at the first collection date, which makes for more accurate smoothing when performing 7-day averages. If the IHE only reports *cumulative* cases or tests for the Fall 2020 semester, we leave the “total_tests” and “positive_tests” columns blank and report the “cumulative_tests” and “cumulative_cases” in the “notes” column, which we extract later in the analyses. **Table C: Description of variables in** Table 1. Where appropriate, we use the “per 100k” designation—the variable’s value divided by county population, multiplied by 100,000. Here “log” refers to the natural log, which we apply to variables that follow heavy-tailed distributions (e.g. income and population density). **Fig A: JSD between distributions of demographic variables**. As we vary the threshold for inclusion into the two groups—counties with IHEs that returned primarily in-person for Fall 2020 and counties with IHEs that remained primarily online—the Jensen-Shannon Divergence also changes. We want to select the value for this threshold based on whatever minimizes the Jensen-Shannon divergence, on average. **Fig B: Comparison of county-level demographics between groups**. Here, we compare the two groups—counties with IHEs that returned primarily in-person for Fall 2020 and counties with IHEs that remained primarily online—based on distributions of **(a)** age, **(b)** race, **(c)** income, and **(d)** urban-rural designation. Error bars: 95% confidence intervals. **Fig C: Map of counties included in matched analysis**. With the exception of California, which includes many primarily online IHEs, there are very few regions where the counties are clustered based on campus reopening strategy. County and state boundary maps downloaded from the United States Census TIGER/Line Shapefiles [52]. **Fig D: Distributions of the variables used in the regression in** Table 1. **Fig E: Example data: Northeastern University**. **Fig F: Example data: North Carolina State University**. **Fig G: Example data: University of California-Los Angeles**. **Fig H: Example data: Purdue University**. **Fig I: Example data: University of Miami**. **Fig J: Example data: Georgia Institute of Technology**. **Fig K: Example data: Duke University**. **Fig L: Example data: Ohio State University**.
(PDF)

## References

[1] Centers for Disease Control and Prevention. Risk for COVID-19 Infection, Hospitalization, and Death By Age Group; 2021. https://www.cdc.gov/coronavirus/2019-ncov/covid-data/investigations-discovery/hospitalization-death-by-age.html.

[2] Boehmer T, DeVies J, Caruso E, van Santen K, Tang S, Black C, et al. Changing Age Distribution of the COVID-19 Pandemic- United States, May-August 2020. Morbidity and Mortality Weekly Report. 2020;69:1404–1409. doi: 10.15585/mmwr.mm6939e1 33001872 PMC7537561

[3] Cunningham J, Vaduganathan M, Claggett B, Jering K, Bhatt A, Rosenthal N, et al. Clinical Outcomes in Young US Adults Hospitalized With COVID-19. JAMA Internal Medicine. 2021;181(3):379–381. doi: 10.1001/jamainternmed.2020.5313 PMC7489373 32902580

[4] Leidman E, Duca LM, Omura JD, Proia K, Stephens JW, Sauber-Schatz EK. COVID-19 Trends Among Persons Aged 0–24 Years—United States, March 1–December 12, 2020. Morbidity and Mortality Weekly Report. 2021;70(3):88–94. doi: 10.15585/mmwr.mm7003e1 33476314 PMC7821770

[5] Goldtein J, Lee R. Demographic perspectives on the mortality of COVID-19 and other epidemics. Proceedings of the National Academy of Sciences. 2020;117(36):22035–22041. doi: 10.1073/pnas.2006392117 PMC7486771 32820077

[6] Latsuzbaia A, Herold M, Bertemes JP, Mossong J. Evolving social contact patterns during the COVID-19 crisis in Luxembourg. PLOS One. 2021;15(8):e0237128. doi: 10.1371/journal.pone.0237128 PMC7410209 32760114

[7] Mossong J, Hens N, Jit M, Beutels P, Auranen K, Mikolajczyk R, et al. Social contacts and mixing patterns relevant to the spread of infectious diseases. PLOS Medicine. 2008;5(3):e74. doi: 10.1371/journal.pmed.0050074 18366252 PMC2270306

[8] Cornwell B. Age trends in daily social contact patterns. Research on Aging. 2011;33(5):598–631. doi: 10.1177/0164027511409442

[9] Hutchins H, Wolff B, Leeb R, Ko J, Odom E, Willey J, et al. COVID-19 Mitigation Behaviors by Age Group–United States, April-June 2020. Morbidity and Mortality Weekly Report. 2020;69(43):1584–1590. doi: 10.15585/mmwr.mm6943e4 33119562 PMC7641002

[10] Hou X, Gao S, Kang Y, Chen N, Chen K, Rao J, et al. Intracounty modeling of COVID-19 infection with human mobility: Assessing spatial heterogeneity with business traffic, age, and race. Proceedings of the National Academy of Sciences. 2021;118(24):e2020524118. doi: 10.1073/pnas.2020524118 PMC8214685 34049993

[11] Monod M, Blenkinsop A, Xi X, Herbert D, Bershan S, Tietze S, et al. Age groups that sustain resurging COVID-19 epidemics in the United States. Science. 2021;371 (6536). doi: 10.1126/science.abe8372 33531384 PMC8101272

[12] Abdullah M, Dias C, Muley D, Shahin M. Exploring the impacts of COVID-19 on travel behavior and mode preferences. Transportation Research Interdisciplinary Perspectives. 2020;8. doi: 10.1016/j.trip.2020.100255 34173481 PMC7640923

[13] Sharangpani R, Boulton K, Wells E, Kim C. Attitudes and behaviors of international air travelers toward pandemic influenza. Journal of Travel Medicine. 2011;18(3):203–208. doi: 10.1111/j.1708-8305.2011.00500.x 21539661

[14] Leggat P, Brown LH, Aitken P, Speare R. Level of concern and precaution taking among Australians regarding travel during pandemic (H1N1) 2009: Results from the 2009 Queensland Social Survey. Journal of Travel Medicine. 2010;17(5):291–295. doi: 10.1111/j.1708-8305.2010.00445.x 20920048

[15] United States Department of Education, National Center for Education Statistics. Total fall enrollment in degree-granting postsecondary institutions, by attendance status, sex of student, and control of institution: Selected years, 1947 through 2029; 2020. https://nces.ed.gov/programs/digest/d20/tables/dt20_303.10.asp.

[16] Leidner AJ, Barry V, Bowen VB, Silver R, Musial T, Kang GJ. Opening of large institutions of higher education and county-Level COVID-19 incidence—United States, July 6—September 17, 2020. Morbidity and Mortality Weekly Report. 2021;70(1):14–19. doi: 10.15585/mmwr.mm7001a4 33411699 PMC7790156

[17] Cai W, Ivory D, Semple K, Smith M, et al. Tracking the Coronavirus at U.S. colleges and universities. New York Times. 2020;.

[18] Nadworny E, McMinn S. Even in COVID-19 hot spots, many colleges aren’t aggressively testing students. NPR. 2020;.

[19] Marsicano CR, Koo D, Rounds E. COVID-19 stats: College and university COVID-19 student testing protocols, by mode of instruction (N = 1,849)—United States, Spring 2021. CDC MMWR. 2021;70(14):535. 10.15585/mmwr.mm7014a5 PMC8030987 33830987

[20] Cheng SY, Wang CJ, Shen ACT, Chang SC. How to safely reopen colleges and universities during COVID-19: experiences from Taiwan. Annals of Internal Medicine. 2020;173(8):638–641. doi: 10.7326/M20-2927 32614638 PMC7339040

[21] Walke HT, Honein MA, Redfield RR. Preventing and responding to COVID-19 on college campuses. JAMA. 2020;17(324):1727–1728. doi: 10.1001/jama.2020.20027 32991681 PMC9648565

[22] Wojtusiak J, Bagchi P, Durbha SSKRTN, Mobahi H, Mogharab Nia R, Roess A. COVID-19 symptom monitoring and social distancing in a university population. Journal of Healthcare Informatics Research. 2021;. doi: 10.1007/s41666-020-00089-x 33437913 PMC7790352

[23] Harris JE. Geospatial analysis of the September 2020 coronavirus outbreak at the University of Wisconsin—Madison: Did a cluster of local bars play a critical role? NBER. 2020;.

[24] Bahl R, Eikmeier N, Fraser A, Junge M, Keesing F, Nakahata K, et al. Modeling COVID-19 spread in small colleges. PLOS ONE. 2021;. doi: 10.1371/journal.pone.0255654 34407115 PMC8372956

[25] Richmond CS, Sabin AP, Jobe DA, Lovrich SD, Kenny PA. SARS-CoV-2 sequencing reveals rapid transmission from college student clusters resulting in morbidity and deaths in vulnerable populations. medRxiv. 2020;.

[26] Yamey G, Walensky RP. COVID-19: Re-opening universities is high risk. BMJ. 2020;370. 32873595 10.1136/bmj.m3365

[27] Perez-Reche F, Strachan N. Estimating the number of COVID-19 cases being introduced into UK Higher Education Institutions during Autumn 2020. medRxiv. 2020;.

[28] Zalesak M, Samaranayake S. SEIR-Campus: Modeling infectious diseases on university campuses. arXiv. 2020;.

[29] Booeshaghi AS, Tan FH, Renton B, Berger Z, Pachter L. Markedly heterogeneous COVID-19 testing plans among US colleges and universities. medRxiv. 2020;.

[30] Andersen MS, Bento AI, Basu A, Marsicano CR, Simon K. College openings in the United States increased mobility and COVID-19 incidence. medRxiv. 2021;. 10.1371/journal.pone.0272820 PMC9423614 36037207

[31] Marinoni G, Van’t Land H, Jensen T. The impact of COVID-19 on higher education around the world. IAU Global Survey Report. 2020;.

[32] Losina E, Leifer V, Millham L, Panella C, Hyle EP, Mohareb AM, et al. College campuses and COVID-19 mitigation: Clinical and economic value. Annals of Internal Medicine. 2020;. doi: 10.7326/M20-6558 33347322 PMC7755069

[33] Floyd DL. 2020, the year none of us predicted: COVID-19 and community colleges. Community College Journal of Research and Practice. 2021;45(1):1–7. doi: 10.1080/10668926.2020.1841649

[34] Poole SF, Gronsbell J, Winter D, Nickels S, Levy R, Fu B, et al. A holistic approach for suppression of COVID-19 spread in workplaces and universities. PLOS ONE. 2021;. doi: 10.1371/journal.pone.0254798 34383766 PMC8360595

[35] Elbanna A, Wong GN, Weiner ZJ, Wang T, Zhang H, Liu Z, et al. Entry screening and multi-layer mitigation of COVID-19 cases for a safe university reopening. medRxiv. 2020;.

[36] Rennert L, Kalbaugh CA, Shi L, McMahan C. Modelling the impact of presemester testing on COVID-19 outbreaks in university campuses. BMJ Open. 2020;10(12). doi: 10.1136/bmjopen-2020-042578 33323447 PMC7745453

[37] Marsicano CR, Felten K, Toledo L, Buitendorp M. Tracking campus responses to the COVID-19 pandemic. APSA Preprints. 2020;.

[38] Bracis C, Burns E, Moore M, Swan D, Reeves DB, Schiffer JT, et al. Widespread testing, case isolation and contact tracing may allow safe school reopening with continued moderate physical distancing: A modeling analysis of King County, WA data. Infectious Disease Modelling. 2021;6:24–35. doi: 10.1016/j.idm.2020.11.003 33294745 PMC7695953

[39] Cai G, Luo S, Zheng X, Yue T, Jin T, Chu X, et al. Safety of reopening universities and colleges using a combined strategy during coronavirus disease 2019 in China: Cross sectional study. SSRN Electronic Journal. 2020; p. 1–16.

[40] Mackert M, Table B, Yang J, Bouchacourt L, Woods JM, Bernhardt JM, et al. Applying best practices from health communication to support a university’s response to COVID-19. Health Communication. 2020;35(14):1750–1753. doi: 10.1080/10410236.2020.1839204 33106047

[41] van Pelt A, Glick HA, Yang W, Rubin D, Feldman M, Kimmel SE. Evaluation of COVID-19 testing strategies for repopulating college and university campuses: A decision tree analysis. Journal of Adolescent Health. 2021;68(1):28–34. doi: 10.1016/j.jadohealth.2020.09.038 33153883 PMC7606071

[42] Borowiak M, Ning F, Pei J, Zhao S, Tung HR, Durrett R. Controlling the spread of COVID-19 on college campuses. Mathematical Biosciences and Engineering. 2020;18(1):551–563. doi: 10.3934/mbe.2021030 33525107

[43] Panovska-Griffiths J, Kerr CC, Stuart RM, Mistry D, Klein DJ, Viner RM, et al. Determining the optimal strategy for reopening schools, the impact of test and trace interventions, and the risk of occurrence of a second COVID-19 epidemic wave in the UK: a modelling study. The Lancet Child & Adolescent Health. 2020;4(11):817–827. doi: 10.1016/S2352-4642(20)30250-9 32758453 PMC7398659

[44] Bergstrom T, Bergstrom CT, Li H. Frequency and accuracy of proactive testing for COVID-19. medRxiv. 2020;.

[45] Centers for Disease Control and Prevention. Considerations for Institutions of Higher Education; 2021. https://www.cdc.gov/coronavirus/2019-ncov/community/colleges-universities/considerations.html.

[46] Centers for Disease Control and Prevention. Colleges, Universities, and Higher Learning Plan, Prepare, and Respond; 2020. https://www.cdc.gov/coronavirus/2019-ncov/community/colleges-universities/index.html.

[47] Althouse BM, Wallace B, Case B, Scarpino SV, Allard A, Berdahl AM, et al. The unintended consequences of inconsistent pandemic control policies. medRxiv. 2020;. doi: 10.1101/2020.08.21.20179473 38798822 PMC11116187

[48] Gumprecht B. The American College Town. Geographical Review. 2003;93(1):51–80. doi: 10.1111/j.1931-0846.2003.tb00020.x

[49] Karmakar M, Lantz P, Tipirneni R. Association of social and demographic factors with COVID-19 incidence and death rates in the US. JAMA Network Open. 2021;4(1):e2036462. doi: 10.1001/jamanetworkopen.2020.36462 33512520 PMC7846939

[50] Jin J, Agarwala N, Kundu P, Harvey B, Zhang Y, Wallace E, et al. Individual and community-level risk for COVID-19 mortality in the United States. Nature Medicine. 2021;27:264–269. doi: 10.1038/s41591-020-01191-8 33311702

[51] Redden E. How Transparent Is Your College’s COVID Dashboard? Inside Higher Ed. 2020. https://www.insidehighered.com/news/2020/10/08/many-colleges-publish-covid-dashboards-theres-no-uniform-standard-public-reporting.

[52] Bureau USC. TIGER/Line Shapefiles—2018; 2018. Available from: https://www.census.gov/geographies/mapping-files.html.

[53] Klein B. jkbren/campus-covid: campus-covid; 2021.

[54] Yanez ND, Weiss NS, Romand JA, Treggiari MM. COVID-19 mortality risk for older men and women. BMC Public Health. 2020;20(1):1–7. doi: 10.1186/s12889-020-09826-8 33213391 PMC7675386

[55] Ahrenfeldt LJ, Otavova M, Christensen K, Lindahl-Jacobsen R. Sex and age differences in COVID-19 mortality in Europe. Wiener klinische Wochenschrift. 2021;133(7):393–398. doi: 10.1007/s00508-020-01793-9 33351155 PMC7755064

[56] Georgetown University Center for Global Health Science and Security, Talus Analytics. The COVID Analysis and Mapping of Policies; 2020. https://www.covidlocal.org/amp.

[57] Hale T, Angrist N, Goldszmidt R, Kira B, Petherick A, Phillips T, et al. A global panel database of pandemic policies (Oxford COVID-19 Government Response Tracker). Nature Human Behaviour. 2021;5(4):529–538. doi: 10.1038/s41562-021-01079-8 33686204

[58] MassGIS (Bureau of Geographic Information). MassGIS Data: Municipalities; 2021. Available from: https://www.mass.gov/info-details/massgis-data-municipalities.

[59] Massachusetts Department of Health. COVID-19 Response Reporting; 2021. https://www.mass.gov/info-details/covid-19-response-reporting.

[60] Boston Globe. UMass Amherst under lockdown because of coronavirus spike; 2021. https://www.bostonglobe.com/2021/02/08/nation/umass-amherst-under-lockdown-due-coronavirus-spike/.

[61] NBC Boston. Amid COVID outbreak, UMass Amherst prohibits students from leaving dorms for walks; 2021. https://www.nbcboston.com/news/local/amid-covid-outbreak-umass-amherst-prohibits-students-from-leaving-dorms-for-walks/2299189/.

[62] Haug N, Geyrhofer L, Londei A, Dervic E, Desvars-Larrive A, Loreto V, et al. Ranking the effectiveness of worldwide COVID-19 government interventions. Nature Human Behaviour. 2020;4(12):1303–1312. doi: 10.1038/s41562-020-01009-0 33199859

[63] Brauner JM, Mindermann S, Sharma M, Johnston D, Salvatier J, Gavenčiak T, et al. Inferring the effectiveness of government interventions against COVID-19. Science. 2021;371 (6531). doi: 10.1126/science.abd9338 33323424 PMC7877495

[64] Bo Y, Guo C, Lin C, Zeng Y, Li HB, Zhang Y, et al. Effectiveness of non-pharmaceutical interventions on COVID-19 transmission in 190 countries from 23 January to 13 April 2020. International Journal of Infectious Diseases. 2021;102:247–253. doi: 10.1016/j.ijid.2020.10.066 33129965 PMC7598763

[65] Perra N. Non-pharmaceutical interventions during the COVID-19 pandemic: A review. Physics Reports. 2021;. doi: 10.1016/j.physrep.2021.02.001 33612922 PMC7881715

[66] Centers for Disease Control and Prevention. Interim Guidance for SARS-CoV-2 Testing and Screening at Institutions of Higher Education (IHEs); 2021. https://www.cdc.gov/coronavirus/2019-ncov/community/colleges-universities/ihe-testing.html.

[67] Gibson GC, Weitz JS, Shannon MP, Holton B, Bryksin A, Liu B, et al. Surveillance-to-diagnostic testing program for asymptomatic SARS-CoV-2 infections on a large, urban campus—Georgia Institute of Technology, Fall 2020. Epidemiology. 2022;33(2):209–216. doi: 10.1097/EDE.0000000000001448 34860727

[68] Stubbs CW, Springer M, Thomas TS. The impacts of testing cadence, mode of instruction, and student density on Fall 2020 COVID-19 rates on campus. medRxiv. 2020;.

[69] Plante JA, Mitchell BM, Plante KS, Debbink K, Weaver SC, Menachery VD. The variant gambit: COVID’s next move. Cell Host & Microbe. 2021;. doi: 10.1016/j.chom.2021.02.020 PMC7919536 33789086

[70] Troiano G, Nardi A. Vaccine hesitancy in the era of COVID-19. Public Health. 2021;. doi: 10.1016/j.puhe.2021.02.025 33965796 PMC7931735

[71] New York Times. See How Vaccinations Are Going in Your County and State; 2021. https://www.nytimes.com/interactive/2020/us/covid-19-vaccine-doses.html.

[72] Dong E, Du H, Gardner L. An interactive web-based dashboard to track COVID-19 in real time. The Lancet Infectious Diseases. 2020;20(5):533–534. doi: 10.1016/S1473-3099(20)30120-1 32087114 PMC7159018

[73] United States Census Department. American Community Survey; 2018. https://www.census.gov/programs-surveys/acs.

[74] United States Department of Education. National Center for Education Statistics, Integrated Postsecondary Education Data System (IPEDS); 2018. https://nces.ed.gov/ipeds/use-the-data.

[75] Smith TP, Flaxman S, Gallinat AS, Kinosian SP, Stemkovski M, Unwin HJT, et al. Temperature and population density influence SARS-CoV-2 transmission in the absence of nonpharmaceutical interventions. Proceedings of the National Academy of Sciences. 2021;118(25). doi: 10.1073/pnas.2019284118 34103391 PMC8237566

[76] Green J, Edgerton J, Naftel D, Shoub K, Cranmer SJ. Elusive consensus: Polarization in elite communication on the COVID-19 pandemic. Science Advances. 2020;6(28). doi: 10.1126/sciadv.abc2717 32923600 PMC7455486

[77] Zurn P, Bassett DS, Rust NC. The citation diversity statement: A practice of transparency, a way of life. Trends in Cognitive Sciences. 2020;24(9):669–672. doi: 10.1016/j.tics.2020.06.009 32762966

[78] Dworkin JD, Linn KA, Teich EG, Zurn P, Shinohara RT, Bassett DS. The extent and drivers of gender imbalance in neuroscience reference lists. Nature Neuroscience. 2020;23(8):918–926. doi: 10.1038/s41593-020-0658-y 32561883

[79] Chakravartty P, Kuo R, Grubbs V, McIlwain C. #CommunicationSoWhite. Journal of Communication. 2018;68(2):254–266. doi: 10.1093/joc/jqy003

[80] Maliniak D, Powers R, Walter BF. The gender citation gap in international relations. vol. 67. Cambridge University Press; 2013.

[81] Dion ML, Sumner JL, Mitchell SML. Gendered citation patterns across political science and social science methodology fields. Political Analysis. 2018;26(3):312–327. doi: 10.1017/pan.2018.12

[82] Caplar N, Tacchella S, Birrer S. Quantitative evaluation of gender bias in astronomical publications from citation counts. Nature Astronomy. 2017;1. doi: 10.1038/s41550-017-0141

[83] Azoulay P, Lynn F. Self-citation, cumulative advantage, and gender inequality in science. Sociological Science. 2020;7:152–186. doi: 10.15195/v7.a7

[84] Ghiasi G, Mongeon P, Sugimoto CR, Larivière V. Gender homophily in citations. 23rd International Conference on Science and Technology Indicators (STI 2018). 2018; p. 1519–1525.

